# SlumberNet: deep learning classification of sleep stages using residual neural networks

**DOI:** 10.1038/s41598-024-54727-0

**Published:** 2024-02-27

**Authors:** Pawan K. Jha, Utham K. Valekunja, Akhilesh B. Reddy

**Affiliations:** 1 Department of Systems Pharmacology and Translational Therapeutics, Perelman School of Medicine, University of Pennsylvania, Philadelphia, PA 19104 USA; 2 Institute for Translational Medicine and Therapeutics, Perelman School of Medicine, University of Pennsylvania, Philadelphia, PA 19104 USA; 3 Chronobiology and Sleep Institute (CSI), Perelman School of Medicine, University of Pennsylvania, Philadelphia, PA 19104 USA

**Keywords:** Computational biology and bioinformatics, Neuroscience

## Abstract

Sleep research is fundamental to understanding health and well-being, as proper sleep is essential for maintaining optimal physiological function. Here we present SlumberNet, a novel deep learning model based on residual network (ResNet) architecture, designed to classify sleep states in mice using electroencephalogram (EEG) and electromyogram (EMG) signals. Our model was trained and tested on data from mice undergoing baseline sleep, sleep deprivation, and recovery sleep, enabling it to handle a wide range of sleep conditions. Employing k-fold cross-validation and data augmentation techniques, SlumberNet achieved high levels of overall performance (accuracy = 97%; F1 score = 96%) in predicting sleep stages and showed robust performance even with a small and diverse training dataset. Comparison of SlumberNet's performance to manual sleep stage classification revealed a significant reduction in analysis time (~ 50 × faster), without sacrificing accuracy. Our study showcases the potential of deep learning to facilitate sleep research by providing a more efficient, accurate, and scalable method for sleep stage classification. Our work with SlumberNet further demonstrates the power of deep learning in mouse sleep research.

## Introduction

Sleep research is critical for understanding health and well-being, as sleep plays a vital role in numerous physiological processes. These processes include memory consolidation, cognitive function, immune system regulation, cellular repair, and hormonal balance. Proper sleep is essential for maintaining optimal physical and mental health, and disruptions in sleep can lead to a wide range of adverse effects^1^. Sleep disturbances, such as insomnia, sleep apnea, and circadian rhythm disorders, are increasingly prevalent in modern society, affecting millions of people worldwide^2^. Chronic sleep disruptions have been linked to an increased risk of developing various health conditions, including obesity, diabetes, cardiovascular disease, and even certain types of cancer^3^. By studying sleep patterns and their underlying mechanisms, we can gain valuable insights into the importance of sleep and develop novel interventions to address sleep-related disorders.

Mice serve as essential model organisms for investigating sleep patterns and their implications in humans^4^. Sleep stages in mice can be categorized into wake, non-REM (NREM), and REM stages^5–7^. During the wake stage, mice exhibit active brain and body functions, resulting in mixed frequency electroencephalography (EEG) signals and large electromyography (EMG) amplitudes^8^. The NREM stage, accounting for over 90% of sleep, is marked by cortical synchronization in the brain and a resting body, leading to lower peak EEG frequencies, higher EEG amplitudes, and smaller EMG amplitudes. The REM stage is characterized by active brain function and a motionless body, as evidenced by low EMG amplitudes^9,10^. Classification of wake and sleep periods into these three stages based on EEG and EMG signals is a time-consuming part of sleep analysis, since many hours (or days) or data need to be analysed by visual inspection of the data.

In this paper, we present a novel deep learning model (“SlumberNet”) based on the residual network (ResNet) architecture^11^, specifically designed for the classification of sleep states in mice using EEG and EMG signals. Our goal is to advance the understanding of sleep and its underlying mechanisms by leveraging the power of deep learning to vastly speed up sleep stage classification. We assessed both mice undergoing baseline (undisturbed sleep) and mice subjected to sleep deprivation for 12 h. This approach allowed us to train and test the SlumberNet model in perturbed states, which, to our knowledge, has not been done before.

Several sleep scoring methods for mice have been put forward before^12–19^. Deep learning has made significant strides in recent years, particularly in image classification, where convolutional neural networks (CNNs) have demonstrated exceptional performance^20^, including in sleep stage classification using large datasets^5^. Residual Networks (ResNet) have emerged as a prominent CNN architecture, addressing the vanishing gradient problem associated with training deeper models^21^. Inspired by these developments, we adapted the ResNet architecture to analyze time-series data, such as EEG and EMG signals, to classify sleep states in mice. By leveraging a smaller but diverse dataset of mice, we were able to validate SlumberNet's effectiveness and robustness against individual differences and noise.

## Results

### Data preprocessing and validation of EEG and EMG signals and spectra for training

**Figure 1:**
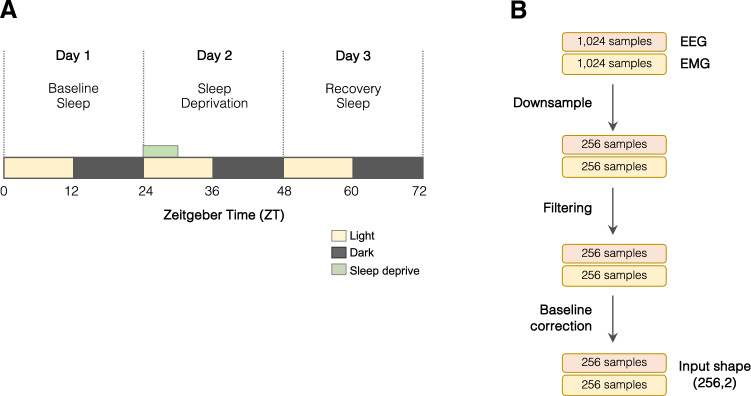
Experimental protocol, data acquisition, and preprocessing for input into SlumberNet. (**A**) Experimental protocol to record EEG and EMG signals for three successive days. Animals were maintained in 12 h:12 h light: dark cycles throughout. ZT0 indicates the onset of the first light phase; ZT12 indicates onset of the first dark phase. On the first day, animals were allowed to sleep ad libitum. On the second day, animals underwent 6 h of sleep deprivation in the first half of the light phase by gentle handling. Mice were subsequently allowed to sleep ad libitum on that day and beyond. (**B**) Schematic showing preprocessing steps performed on EEG and EMG data that has been sleep-staged in 4-s epochs. Data (256 Hz) were scored in 4-s epochs (1024 samples per epoch). Paired EEG and EMG data for each epoch were downsampled to 64 Hz so that there were 256 samples per 4-s epoch. Data were filtered (EMG only) using a Butterworth filter and then baseline drift was corrected by subtracting the baseline from the raw data. The shape of the combined input array for the deep learning model was (256, 2).

We acquired data from a group of C57BL/6J mice in order to build a training set for the SlumberNet model. As well as being able to analyze baseline EEG and EMG signals, we wanted to increase SlumberNet’s potential utility by training it on data from animals undergoing sleep deprivation and recovery sleep. This would allow the model to handle signals from a variety of sleep paradigms that are typically incorporated into sleep analyses. Therefore, we designed a protocol encompassing all of these elements over three successive days (Fig. 1A).

We acquired telemetric EEG and EMG signals (bipotential electrodes) and exported the data at a frequency of 500 Hz. To classify sleep stages for the 72 h time course for each animal, we downsampled the data to 256 Hz and then imported into SleepSign software for manual sleep stage classification. The data were split into 4-s epochs and each of epoch was classified as Wake (W), NREM (N), REM (R), or artefact. Artefact was attributable to movement artefact, and thus excluded from the training dataset in preprocessing (see Methods). We also excluded the entire dataset from any animal that was marred by significant electrical noise or artefact, as this was difficult to classify manually, and data from such animals would be excluded from analysis in our normal (manual) analysis workflow.

To preprocess the data for inputting into the model, we split all of the animal datasets so that we obtained matching EEG, EMG, and sleep stage data for each 4 s epoch (1,024 EEG/EMG samples at 256 Hz). To decrease the total number of model parameters while retaining essential features of the data, we further downsampled the data fourfold, such that 1,024 samples became 256 samples per 4 s epoch. The EMG signal was then put through a Butterworth filter to cut out significant electrical noise, and then both EEG and EMG signals were baseline-corrected to remove any time-dependent drift in the signals (Fig. 1B). Thus, the final electrical data that were inputted into the model consisted of an array 256 EEG and 256 EMG voltages for each 4 s epoch and a matched sleep stage for that epoch. The sleep stage data were one-hot encoded (Wake = [1,0,0]; NREM = [0,1,0]; REM [0,0,1]) and the goal of the model (its output) was to provide an array of three values (between 0 and 1) that indicates a probability given the EEG/EMG array as input.

**Figure 2:**
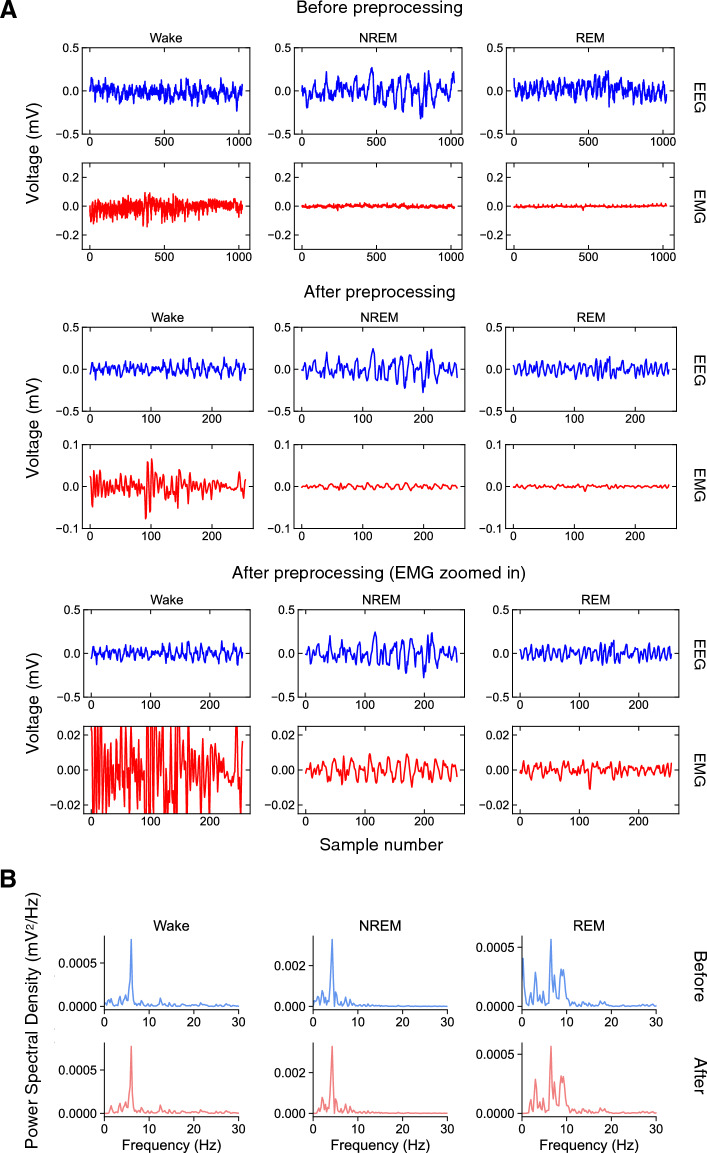
Examples of preprocessing steps for training. (**A**) Examples of wake, NREM, and REM EEG and EMG traces for a 4-s epoch. The traces are shown before (top) and after (middle) preprocessing has been performed. The bottom trace shows a zoomed-in y-axis for EMG traces to illustrate the amplitude differences between the three sleep stages. (**B**) Power spectral density (PSD) for the different sleep stages before and after preprocessing.

We provide an example of 256 Hz data that was used for manual sleep stage classification, and the effect of preprocessing steps on the data (Fig. 2A). The features of the data remain intact, including voltage amplitudes and frequencies, and also the spectral densities of the EEG signal (Fig. 2B). Thus, preprocessing the data for input into the model did not lead to significant loss of information used to classify the EEG and EMG signals successfully both by visual inspection and spectral metrics.

### SlumberNet model architecture

**Figure 3:**
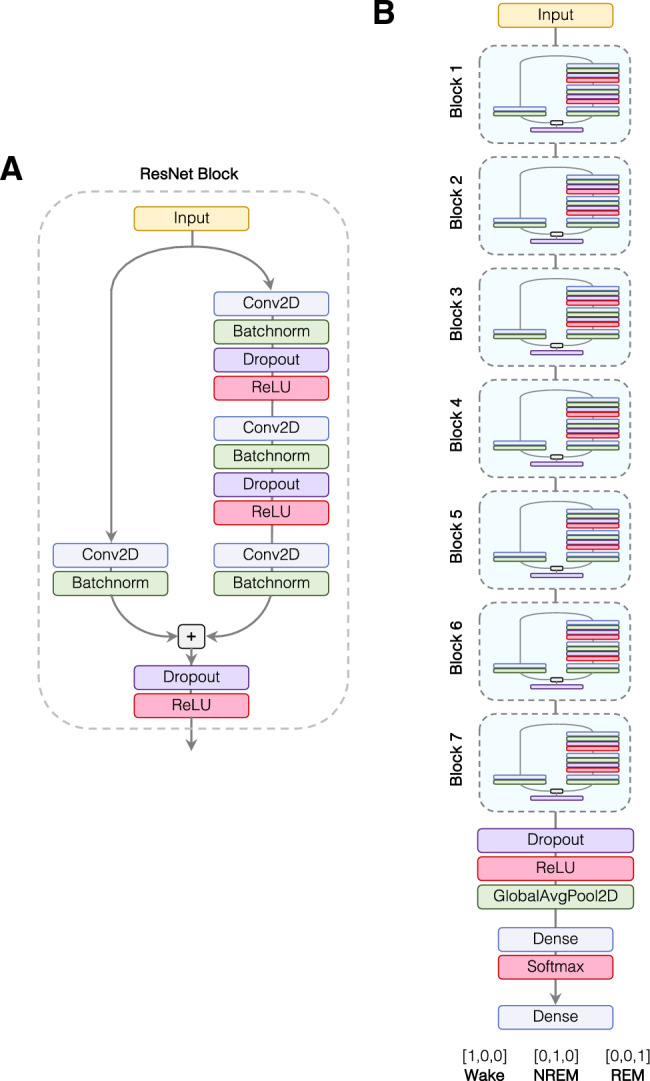
SlumberNet model schematic. (**A**) Single ResNet block architecture: input data (2 × 256 samples: 1 × 256 EEG and 1 × 256 EMG) undergo processing by a 2D convolutional layer (Conv2D), followed by batch normalization (BatchNorm) and optional dropout. A rectified linear unit (ReLU) serves as the activation layer. This sequence is repeated before a final Conv2D layer is combined with the input via a shortcut connection in the ResNet block, ending with an activation layer. (**B**) Complete SlumberNet model: input data pass through seven successive ResNet blocks with filter numbers doubling at each step. A global average pooling layer precedes a dense (fully connected) softmax-activated layer, yielding three probabilities for classification: Wake ([1,0,0]), NREM ([0,1,0]), or REM ([0,0,1]).

We built a ResNet model based on a 2-dimensional convolutional layer (Conv2D) that could take both the EEG and EMG signals as input simultaneously. The first dimension represents the time-ordered data of the voltage signal for the epoch, and the second dimension contains the signal type (EEG or EMG). This setup ensures that EEG and EMG signals are temporally matched to each other. We adopted this approach in case there are latent representations in the EMG signal that are not simply related to the absolute values (amplitude) of the EMG. Each block contains a variety of other layers including batch normalization (BatchNorm) and Dropout (Fig. 3A), although we did not need to use the dropout option as we did not observe significant overfitting in our overall model.

To produce the final model, we experimented with various numbers of concatenated ResNet blocks to balance the overall number of training parameters (which increases complexity and training time) and overfitting. We settled on seven blocks in total (Fig. 3B). This model did not overfit to the training data and thus produced validation accuracies similar training accuracies. Fewer blocks could not represent the dataset fully and accuracies failed to reach 97% (which was achieved with seven blocks). A greater number of blocks resulted in longer training times, and also overfitting to the training data without improvement in validation accuracy (which peaked at ~ 97%). Such models are not useful as they will not generalize to new data that are not within the training dataset (as shown by the plateauing of validation accuracies). We did not attempt to optimize other hyperparameters (e.g. convolutional filter size, number of filters etc.).

The final layers of the model after the ResNet blocks led to global average pooling and then output of the model through a Softmax layer (normalized exponential function) to yield a final dense (fully-connected) layer of three values that represent the prediction of the sleep stage represented by the input EEG and EMG data for a 4 s epoch (Fig. 3B).

### Training and validation of SlumberNet on mouse EEG and EMG data

We designed our model to detect features purely in the EEG and EMG voltage data in a 4 s epoch, which enabled it to classify sleep stages without any information about classifications of the surrounding sleep epochs. To ensure that this setup was maintained during training, we shuffled the epochs so that the temporal order was randomized across the entire dataset. We employed a training/validation split of the training data, so that 80% of sleep samples were used for training, and the remaining 20%, which were never seen by the model for training, were used to check the accuracy of the model at each training step (training epoch).

In addition, we also used data augmentation methods to increase the generalization of the model, and to avoid overfitting. Our data augmentation involved translating the EEG and EMG data in a 4 s rolling window by a random amount. We also added random amplitude adjustments to each datapoint and added Gaussian noise. This ensured that the data fed into the model in each training epoch was different in a random way. The validation data were not augmented to ensure that the validation accuracy was consistent and comparable between training epochs.

To verify the robustness of the model we used k-fold (fivefold) cross-validation, which is a standard method used in machine learning replication^22^. To do this, the entire dataset was split into five equal training and testing sets, such that the testing set (20% of total) each time was different to any other testing set. The remainder of sleep samples was allocated for training in each set. We ensured that there was an equal number of each type of epoch in each of the five folds (to ensure that training and testing sets were equally balanced each time). This was important since the overall proportions of Wake, NREM and REM sleep stages are different, with Wake epochs predominating. If we did not do this, the model would not generalize well because of imbalances in the training (and testing) sets between folds.

**Figure 4:**
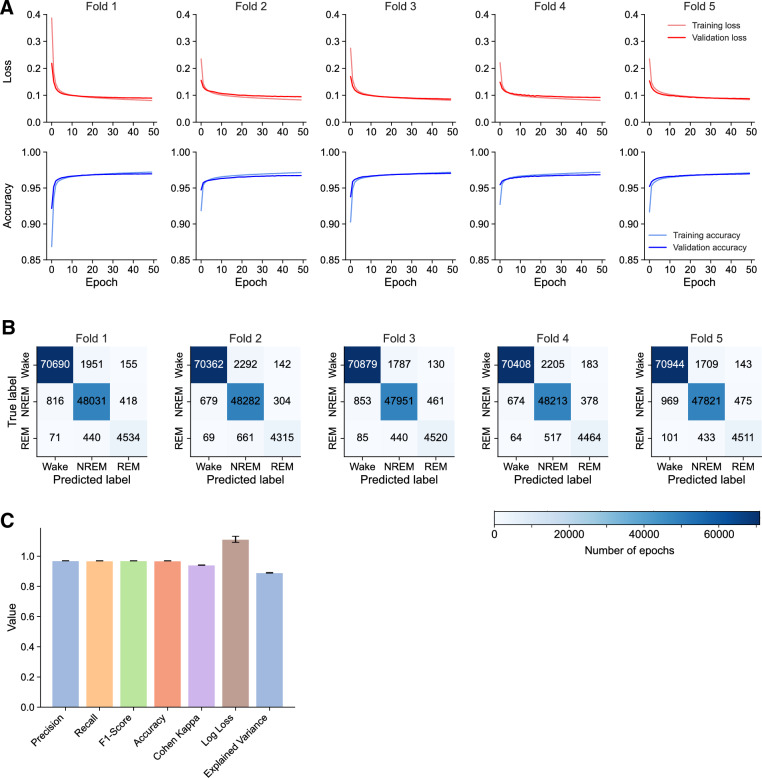
Training and validation of SlumberNet using k-fold (fivefold) cross-validation. (**A**) Plots showing training and validation loss and accuracy metrics for each fold of cross-validation. (**B**) Confusion matrices showing relationships between predicted and true labels of sleep stages. Numbers represent the absolute number of epochs assessed in the validation phase of each training fold. (**C**) Combined validation metrics for all folds in cross-validation (precision, recall, F1-score, accuracy, Cohen kappa, log loss and explained variance). Data are value ± SEM (n = 5 training folds).

Each of the five folds was consistent, with little variability between them (Fig. 4A). In all, there was excellent agreement between training and validation accuracies (and loss), with no evidence of significant overfitting up to 50 epochs of training. Confusion matrices comparing the true and predicted sleep stages for the input data in each fold showed agreement, with the proportions of sleep stages being the same (Fig. 4B). Overall, metrics such as precision and accuracy were very similar between folds (Fig. 4C and Table 1). These data show that our model predicted Wake and NREM stages better than REM (Table 1), which is comparable with other convolutional neural networks built with larger training datasets (Table 2)^5^. Together, these data show that our model is capable of generalizable behavior even with a small training dataset.

**Table 1:** Accuracy, precision, recall, F1 score, Cohen’s kappa (*κ*) metrics for Wake, NREM, and REM sleep samples in each cross-validation fold.

Fold	Wake					NREM					REM				
	Accuracy (%)	Precision (%)	Recall (%)	F1 score (%)	Cohen's *κ*	Accuracy (%)	Precision (%)	Recall (%)	F1 score (%)	Cohen's *κ*	Accuracy (%)	Precision (%)	Recall (%)	F1 score (%)	Cohen's *κ*
1	97.65	98.76	97.11	97.93	0.9520	97.15	95.26	97.50	96.36	0.9402	99.15	88.78	89.87	89.32	0.8888
2	97.50	98.95	96.66	97.79	0.9491	96.90	94.24	98.00	96.08	0.9352	99.07	90.63	85.53	88.01	0.8753
3	97.75	98.69	97.37	98.03	0.9542	97.21	95.56	97.33	96.44	0.9415	99.12	88.44	89.59	89.01	0.8855
4	97.54	98.96	96.72	97.83	0.9499	97.03	94.66	97.86	96.23	0.9378	99.10	88.84	88.48	88.66	0.8819
5	97.70	98.51	97.46	97.98	0.9531	97.18	95.71	97.07	96.39	0.9407	99.09	87.95	89.42	88.68	0.8820
Mean	**97.63**	**98.78**	**97.06**	**97.91**	**0.9517**	**97.10**	**95.08**	**97.55**	**96.30**	**0.9391**	**99.11**	**88.93**	**88.58**	**88.74**	**0.8827**
SEM	0.04	0.07	0.13	0.04	0.0008	0.05	0.23	0.14	0.05	0.0009	0.01	0.37	0.65	0.18	0.0018

Data are percentages (except Cohen’s *κ*) for each fold, and mean (bold typeface) and standard error of the mean (SEM) data are shown for all folds.

**Table 2:** Classification metrics for Wake, NREM and REM sleep samples in difference machine learning models for sleep stage classification.

Model	N	Wake		NREM		REM		Recall (%)	Precision (%)	Accuracy (%)	F1 score (%)	Cohen’s *κ*
		Recall (%)	Precision (%)	Recall (%)	Precision (%)	Recall (%)	Precision (%)					
Models using small numbers of animals for training												
SlumberNet	**9**	**97.1**	**98.8**	**97.6**	**95.1**	**88.6**	**88.9**	**97.9**	**94.2**	**97.0**	**96.0**	**0.94**
MC-SleepNet	14	99.3	98.6	93.9	99.6	99.5	62.6	97.3	86.7	96.4	91.7	0.94
Random Forest	14	95.9	92.6	94.3	95.6	73.9	88.9	88.3	92.6	94.0	90.4	0.89
FASTER	14	89.5	–	94.2	–	78.4	–	–	–	91.1	–	–
MASC	14	95.5	97.3	94.7	96.9	94.0	65.8	94.8	86.7	95.0	90.6	0.91
LSTM model	14	96.2	94.8	95.1	95.8	82.1	85.2	91.5	92.3	94.9	91.9	0.91
Models using large numbers of animals for training												
MC-SleepNet	4200	98.1	97.8	95.8	97.3	80.1	90.1	91.6	95.4	96.7	93.5	0.94

Overall recall, precision, accuracy, F1 score and Cohen’s kappa coefficient for all epochs is shown for each model. Values in bold are for SlumberNet. The large MC-SleepNet model using N = 4200 animals is also shown for comparison.

### Validation of SlumberNet on previously unseen data

To test how well SlumberNet performed on newly-acquired data, we trained the model on all of the training data. We chose the final model (after 50 training epochs) to perform further work on. We performed a new set of experiments using the same protocol as before on n = 3 C57BL/6 J mice (see Fig. 1A). We preprocessed data in the same way as for training, and then inputted the data into the final model and recorded the output for each sleep sample. For each 72 h time course, inference using a single graphics processing unit (GPU) took ~ 1 h. In parallel, we manually scored each 4 s EEG/EMG epoch. In comparison, manual scoring for each animal took ~ 48 h of continuous work, or ~ 6 working days for a typical researcher.

**Figure 5:**
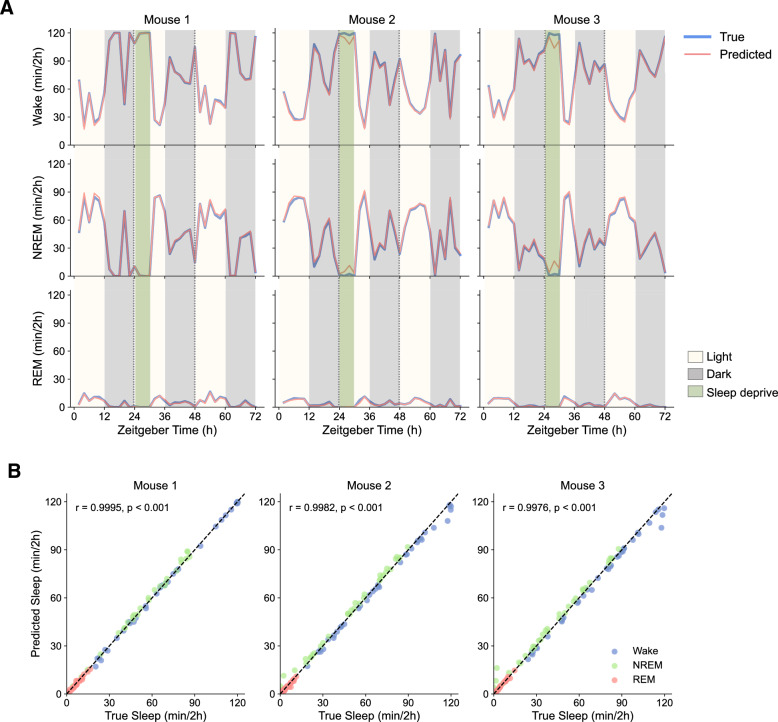
Validation of SlumberNet in experimental data not present in the training set. (**A**) Comparison of manually-scored sleep stage classification (True) vs model-predicted (Predicted) classification by SlumberNet. Each type of sleep stage (Wake, NREM, REM) is shown separately. The three mice were maintained in 12 h:12 h light:dark cycles throughout the assessments and underwent 6 h sleep deprivation on the second day (from ZT24-30; green shading). Data are plotted using 2 h binning of data to calculate the number of minutes of each sleep stage per 2 h bin. (**B**) Correlation of True and Predicted sleep stage amount (minutes per 2 h) in each of the three mice shown in (**A**). Sleep stages are color-coded. Pearson correlation coefficients (r) and associated p-values are shown for each mouse.

We then compared the SlumberNet-predicted sleep stages (Wake, NREM, REM) with those obtained by using the final model’s inference (Fig. 5). There was excellent concordance between the time course data for all of the mice that we tested (Fig. 5A), with baseline sleep, sleep deprivation, and recovery sleep mapping to each other almost perfectly. Accordingly, correlation coefficients for all sleep stages were almost unity (Pearson r = 0.9984 ± 0.001; mean ± standard deviation), and other overall metrics were very similar to those obtained during training and testing (compare Tables 1 and 3). The only significant divergence was in the classification of Wake and NREM sleep stages, which were under- or over-predicted (respectively) by SlumberNet in Mouse 2 and Mouse 3, but not in Mouse 1 (Fig. 5A,B; Table 3).

**Table 3:** Accuracy, precision, recall, F1 score, Cohen’s kappa (*κ*) metrics for Wake, NREM, and REM sleep samples for unseen validation data from n = 3 mice.

Mouse	Wake					NREM					REM				
	Accuracy (%)	Precision (%)	Recall (%)	F1 score (%)	Cohen's *κ*	Accuracy (%)	Precision (%)	Recall (%)	F1 score (%)	Cohen's *κ*	Accuracy (%)	Precision (%)	Recall (%)	F1 score (%)	Cohen's *κ*
1	98.33	99.04	98.23	98.63	0.9650	97.75	95.65	97.94	96.78	0.9505	99.19	93.66	86.33	89.85	0.8942
2	97.71	99.38	96.70	98.03	0.9531	97.08	93.92	98.66	96.23	0.9385	99.17	91.99	83.38	87.47	0.8704
3	97.35	99.29	96.36	97.80	0.9446	96.82	93.20	98.20	95.64	0.9314	99.39	92.04	89.36	90.68	0.9036
Mean	**97.80**	**99.24**	**97.10**	**98.15**	**0.9542**	**97.22**	**94.26**	**98.27**	**96.22**	**0.9401**	**99.25**	**92.56**	**86.35**	**89.33**	**0.8894**
SEM	0.29	0.10	0.58	0.25	0.0059	0.28	0.73	0.21	0.33	0.0056	0.07	0.55	1.73	0.96	0.0099

Data are percentages (except Cohen’s *κ*) for each fold, and mean (bold typeface) and standard error of the mean (SEM) data are shown for each mouse.

### Comparison of SlumberNet to other automated scoring methods

We compared SlumberNet’s performance with a set of automated scoring methods that use small numbers of mice to train their models: MC-SleepNet^5^, Random Forest^5^, FASTER^5,13^, MASC^5^, and LSTM^5^ (Table 2). This reveals an appreciable difference in the efficacy of various sleep stage scoring methods. SlumberNet, which is trained on a modest dataset of nine animals, demonstrates striking precision and accuracy. The model achieves a κ coefficient of 0.94, signifying a high degree of concordance between the model's scores and those from expert manual scoring. SlumberNet exhibits high recall rates in discerning Wake, NREM, and REM sleep stages, achieving 97.6%, 97.1%, and 99.1% respectively. Moreover, it yields a remarkably high precision rate of 98.8% for the Wake stage. In this context, recall pertains to the proportion of actual positive cases accurately identified, while precision refers to the proportion of predicted positive cases correctly classified. Hence, these metrics highlight SlumberNet's prowess in accurate identification and correct classification of each sleep stage.

SlumberNet overall achieves performance on par with, or better than, other existing models used for mouse sleep scoring. Despite using fewer animals for training compared to models such as MC-SleepNet, Random Forest, FASTER, MASC, and a LSTM model, SlumberNet maintains a high degree of scoring accuracy, thereby demonstrating its efficiency and robust learning capacity (Table 2). A salient feature that potentially sets SlumberNet apart from these other models is its use of data augmentation techniques during training. Data augmentation, which refers to the creation of new data based on modifications of the existing dataset, enhances the model's ability to generalize and cope with the diversity of the unseen data, thereby bolstering its performance. This could explain SlumberNet's remarkable precision in Wake stage scoring and high recall in REM stage scoring, as it allows for the model to better capture the nuances in the sleep architecture of mice. Consequently, it is likely that SlumberNet's application of advanced feature extraction, scoring phases, and data augmentation practices culminate in maximizing the detection of salient sleep stage information from EEG and EMG signals.

## Discussion

Our research introduces SlumberNet, a deep learning model rooted in the ResNet architecture, tailored for differentiating sleep stages in mice through EEG and EMG readings. Demonstrating impressive accuracy on a limited dataset, SlumberNet underscores its potential to deepen our insights into sleep dynamics and broaden avenues for sleep disorder research. A standout feature is its ability to generalize to unencountered data, consistently aligning with manual sleep stage assessments, and streamlining the time-intensive sleep analysis process.

**Figure 6:**
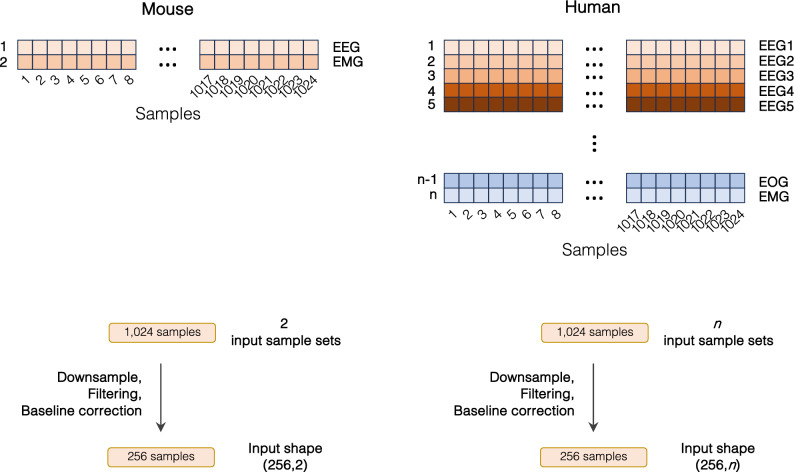
Schematic showing how SlumberNet can be adapted to human polysomnographic data as input. Mouse data with 2 input sample sets (EEG and EMG) are downsampled and inputted into the model as shown. Similarly, human sample sets consisting of *n* input sample sets (EEG channel 1, EEG channel 2… EOG (electrooculogram), EMG) are identically processed and inputted into the model with shape (256,*n*) rather than (256,2) for mouse data.

Given the documented similarities in sleep behaviors between mice and humans^23^, there is a promising avenue for SlumberNet's extension to human EEG sleep diagnostics, as delineated in Fig. 6. Such an adaptation could enhance the precision and efficiency of human sleep pattern analytics, opening doors for tailor-made sleep interventions and a refined understanding of sleep anomalies.

Nevertheless, it is crucial to address some of our study's constraints. SlumberNet might face challenges with exceptionally noisy datasets, especially those riddled with electrical interference or anomalies. Improved preprocessing methodologies targeting noise elimination could bolster the model's efficacy, as well as additional training with especially noisy datasets. It is also worth noting that our model's foundation on a somewhat restricted dataset might pose questions about its scalability. One limitation of our study that warrants consideration is the method employed for data splitting, specifically not partitioning the data by individual mouse subject-ID. We split data en masse to ensure balanced sample classes (Wake, NREM, REM) in k-fold analyses, consistent with previous studies. The absence of this stratification could potentially introduce bias, as recordings from the same mouse might exhibit inherent similarities that could influence the model's validation performance. However, we did not observe this in our validation tests on completely unseen data, but it remains a theoretical concern. Recognizing this, future research could explore the impact of incorporating subject ID-based data splitting to better understand its effect on model performance and generalizability. Future endeavors will pivot towards refining SlumberNet with expansive datasets, covering varied species and experimental scenarios, to truly ascertain its adaptability and relevance.

Compared to previous methods, SlumberNet offers several advantages. Firstly, the adaptation of the ResNet architecture for time-series data allows for more effective analysis of EEG and EMG signals, as well as better handling of the vanishing gradient problem associated with training deeper models. This results in improved classification performance and reduced training times. Secondly, the use of data augmentation techniques and cross-validation ensures the robustness of our model, minimizing overfitting and allowing for better generalization to new data. Lastly, the application of our model to sleep-deprived mice demonstrates its versatility and applicability to a wide range of sleep paradigms.

## Conclusion

SlumberNet is a promising tool for delineating sleep stages in mice using EEG and EMG data, showing high accuracy even on limited datasets. Given the time saved by using our model compared to manual scoring (~ 50 × faster, or ~ 150 × faster accounting for the total number of days it would take manually), SlumberNet should make mouse sleep staging far more rapid than manual approaches, without sacrificing accuracy.

## Methods

### Animals

All animal studies adhered to approved guidelines at the University of Pennsylvania, and ARRIVE guidelines. All animal experimental protocols were approved by the Institutional Animal Care and Use Committee (IACUC) at the Perelman School of Medicine at the University of Pennsylvania. We used wild type, male C57BL/6 J mice from Jackson Laboratories and allowed them to acclimate for a minimum of two weeks before experiments. For sleep deprivation experiments, mice were housed individually in automated sleep fragmentation chambers (Model #80391, Campden/Lafayette Instrument Lafayette, IN, USA). At all times, the mice were given ad libitum access to food and water under standard housing conditions, under a 12-h light: 12-h dark cycle.

### Sleep recording and data acquisition

We implanted telemetry transmitters (HD-X02, Data Sciences International, St. Paul, MN, USA) connected to electrodes for continuous EEG/EMG recording in mice aged between 9 and 11 weeks. The mice were anesthetized with isoflurane (induction 3–4%, maintenance 2–2.5%), and two stainless steel EEG electrodes (length of screw shaft: 2.4 mm; head diameter: 2.16 mm; shaft diameter: 1.19 mm; Plastics One, Roanoke, VA, USA) were implanted epidurally over the right frontal and parietal cortices. The electrodes were connected to the telemetry transmitter with medical-grade stainless steel wires and secured with dental cement (Kemdent, Purton, Swindon, UK). Two EMG stainless-steel leads were inserted into the neck muscles ∼ 5 mm apart and sutured in place. The telemetry transmitter was positioned in a subcutaneous pocket on the left dorsal flank. We administered analgesia during the surgery (subcutaneous injection of buprenorphine (Vetergesic) at 0.1 mg/kg and meloxicam (Metacam) at 10 mg/kg) and allowed animals to recover for at least 10 days before starting experimental protocols. We then recorded EEG/EMG signals continuously for 6–7 days using Data Sciences International hardware and Dataquest ART software (Data Sciences International, St. Paul, MN, USA) with a 500 Hz export rate for downstream analysis.

### Sleep stage scoring

Data were downsampled to 256 Hz from 500 Hz and vigilance states were determined using SleepSign for Animal ver. 3 (Kissei Comtec, Nagano, Japan) as detailed previously^24^. Briefly, we performed manual sleep stage scoring in 4-s epochs by waveform and FFT spectrum recognition. Low amplitude EEG and high amplitude EMG signals were considered as Wake. Slow waves and high amplitudes of EEG coupled with low amplitude EMG signals were considered NREM. Low amplitude EEG dominated by theta frequencies (5–9 Hz), and loss of EMG muscle tone was defined as REM. Defined sleep–wake stages were cross-examined and corrected if necessary.

### Sleep deprivation

Sleep deprivations were performed using a device that applied tactile stimulus with a horizontal bar sweeping just above the cage floor (bedding), as described previously^24^. Once the sweeper was on, animals needed to step over it to continue their normal activities. Sleep deprivation began at the start of the light cycle (Zeitgeber Time 0 (ZT0)) and lasted for 12 h, with continuous sweeping mode (approximately 7.5 s cycle time). We made additional attempts to maintain wakefulness during the second half of the sleep deprivation period by occasionally tapping on the cage or gently touching the animals with a brush. To evaluate the effects of 12-h sleep deprivation on sleep/wake behaviors, we recorded baseline EEG/EMG data for three days after mice had acclimated for a week. Mice were then recorded for an additional 3–4 days during the sleep deprivation and recovery phases. After sleep deprivation, animals were allowed to recover for 24 h.

### Data preparation

Initially, we gathered EEG and EMG information from several files, each containing paired raw voltage data and manually identified sleep stages. The raw voltage data included EEG and EMG signals sampled at a rate of 256 Hz. We arranged these files in a Pandas dataframe, ensuring they were in the appropriate format with both voltage columns represented by floating point numbers. Files not conforming to this format were excluded from further analysis. Upon loading the voltage data, we extracted sleep stage labels from the matched sleep scoring files, designating sleep stages as wake (W), NREM sleep (N), or REM sleep (R). Any other labels in the epoch files were considered artifacts and labeled as "A". We then iterated through the epochs, extracting corresponding EEG and EMG voltage data and adding it to the overall data array. Concurrently, we created a matching NumPy array for the epoch labels, employing one-hot encoding for the sleep stages (W: [1,0,0], N: [0,1,0], R: [0,0,1], A: [1,1,1]).

Next, we downsampled the EEG and EMG data and conducted baseline correction for each epoch. The original EEG and EMG data consisted of 1024 samples per epoch, which we reduced by a factor of 4 to achieve 256 samples per epoch. This downsampling was vital for decreasing the computational load while preserving sufficient data for precise sleep stage classification. We utilized the resample_poly method from the SciPy library, which combines polyphase filtering and resampling, to perform the downsampling. This method is preferred over alternatives such as decimate or resample, as it provides superior anti-aliasing properties and enhanced signal preservation. We then applied a high-pass filter to the downsampled EMG signals to eliminate slow drifts and further minimize noise. The filter's cutoff frequency was set at 0.5 Hz, as it effectively removed slow drifts without significantly altering essential signal features. We used the Butterworth filter from the SciPy library for this task, as it provides a smooth, distortion-free response in the passband. Each 4 s epoch data for EEG and EMG were then baseline corrected. Lastly, we discarded epochs labeled as artifacts (A) from both the voltage and epoch arrays. The resulting preprocessed EEG and EMG data, along with the corresponding epoch labels, were saved for later use in training the model.

### Model training and evaluation

We used Keras and Tensorflow2 to construct and train our model. To guarantee reproducibility, we set a random seed and leveraged all available GPUs on our server via the MirroredStrategy approach for distributed training on multiple GPUs. We employed mixed precision computing (float16 and float32) to accelerate training on Nvidia GPUs with a compute capability of 6.0 or higher, as this method uses lower-precision data types for quicker computations without sacrificing accuracy.

We built our 2D ResNet model with seven ResNet blocks, eight feature maps (kernels), and kernel dimensions of (2, 1). The convolutional layers used were Conv2D layers in Keras/Tensorflow. Since the Conv2D layer accommodates multiple input channels (e.g. for multiple colors in a 2D image), the input shape is (X_dimension, Y_dimension, N_channels), i.e. a 3D array. Since our input data is an array of shape (256,2) and only consists of a single channel, the input shape to the Conv2D layer was (256,2,1). The precise code can be inspected further (see Code Availability section). Moreover, Supplementary Table S1 shows details of each layer in the model and the shapes of each layer, and the number of trainable parameters. Although dropout was an option the ResNet blocks, we set the dropout rate to zero, as we did not observe overfitting. Dropout was also set to zero elsewhere. We employed data augmentation to increase the diversity of the training data, enhancing the model's generalization capabilities. We implemented an AugmentDataGenerator class with a custom augmentation function for TensorFlow-based data augmentation during training. The function applied amplitude scaling to the input EEG and EMG data independently, and both signals were translating by a random amount so that they were temporally yoked together. These transformations helped the model to become more robust against variations in signal strength and to learn invariant features across different time shifts. As a result, the risk of overfitting was minimized.

For training, we subjected our dataset to fivefold cross-validation, a technique for evaluating a model's performance by dividing the dataset into five subsets or "folds." The model was trained over 50 epochs with a learning rate of 1e-06 and a per-GPU batch size of 128. We used the Adam optimizer, an adaptive learning rate optimization algorithm, and wrapped it with a Loss Scale Optimizer to avoid multi-GPU crashes caused by underflow in low-precision computations.

We then established a Stratified Shuffle Split for k-fold cross-validation, a technique that maintains the original class distribution within each fold (original proportions of Wake, NREM, and REM classes within each fold and within the training and validation splits), ensuring that each subset is representative of the overall class distribution. This method provides a more robust evaluation of the model's performance. Using the k-fold cross-validation technique, we trained our model and saved the best model for each fold with a ModelCheckpoint callback, which saves the best version of the model based on a specified metric. A ReduceLROnPlateau callback was also applied to decrease the learning rate if training loss did not improve for three consecutive epochs, enabling more fine-grained optimization during training.

### Model hyperparameters for training and testing

We used the following hyperparameters for training and testing the SlumberNet model:

- 1. num_epochs = 50: The number of epochs to train the model.
- 2. learning_rate = 1e−06: The learning rate for the optimizer.
- 3. batch_size_per_gpu = 128: The batch size per GPU used for training.
- 4. optimizer_name = Adam: The name of the optimizer used for training.
- 5. n_resnet_blocks = 7: The number of ResNet blocks in the model.
- 6. n_feature_maps = 8: The number of feature maps in the top layer of the model.
- 7. kernel_y = 2: The y-dimension of the kernel used in the model.
- 8. strides = (1,1): The stride used in the convolutional layers of the model.
- 9. dropout_rate = 0: The dropout rate applied to the convolutional layers of the model.

### Performance metrics

We loaded the best model for each k-fold cross-validation fold, predicted test set class labels, and calculated various evaluation metrics, such as precision, recall, F1-score, accuracy, confusion matrix, kappa, loss, and explained variance, as outlined below.

Precision is a measure of the accuracy of a model's positive predictions, calculated as the proportion of true positives out of the total predicted positives. It is a useful metric when the cost of false positives is high.

$${\text{Precision}}=\frac{\mathrm{True\,Positive}}{\mathrm{True\,Positive}+\mathrm{False\,Positive}}$$

Recall is a measure of a model's ability to identify all relevant instances, calculated as the proportion of true positives out of the total actual positives. It is useful when the cost of false negatives is high.

$${\text{Recall}}=\frac{\mathrm{True\,Positive}}{\mathrm{True\,Positive}+\mathrm{False\,Negative}}$$

The F1-score is a combined metric that balances both precision and recall, giving equal weight to both metrics. It is calculated as the harmonic mean of precision and recall, and provides a good balance between the two metrics when optimizing for both is important.

$${\text{F}}1\mathrm{ \,Score}=2\cdot \frac{{\text{Precision}}\cdot {\text{Recall}}}{{\text{Precision}}+{\text{Recall}}}$$

Accuracy is a measure of the overall correctness of a model's predictions, calculated as the proportion of correct predictions out of the total number of predictions. It is a simple and intuitive metric, but can be misleading when the classes are imbalanced.

$${\text{Accuracy}}=\frac{\mathrm{True\,Positive}+\mathrm{True\,Negative}}{\mathrm{True\,Positive}+\mathrm{True\,Negative}+\mathrm{False\,Positive}+\mathrm{False\,Negative}}$$

Cohen’s Kappa is a metric that measures the agreement between a model's predictions and the true values, taking into account the expected probability of agreement due to chance. It is a useful metric when the classes are imbalanced or when evaluating inter-rater agreement. A Kappa > 0.8 suggests that the scoring results are in nearly perfect agreement^5^.

$${\text{Kappa}}=\frac{{{\text{p}}}_{{\text{o}}}-{{\text{p}}}_{{\text{e}}}}{1-{{\text{p}}}_{{\text{e}}}}$$

where ${p}_{o}$ is the observed agreement and ${p}_{e}$ is the chance agreement.

Loss is a metric that quantifies the difference between a model's predicted output and the actual output for a given input. It is typically used during the training phase to optimize the model's parameters by minimizing the value of the loss function.

$${\text{Loss}}=\frac{1}{{\text{N}}}{\sum }_{{\text{i}}=1}^{{\text{N}}}L\left({{\text{y}}}_{{\text{i}}},\widehat{{{\text{y}}}_{{\text{i}}}}\right)$$

where $N$ is the number of samples, ${y}_{i}$ is the true value of the $i$th sample, and $\widehat{{y}_{i}}$ is the predicted value of the $i$th sample, and $L$ represents the loss function used in the deep learning model. We used Categorical Cross-Entropy for our loss function, which is defined by:

$$L\left(y,\widehat{y}\right)=-{\sum }_{i=1}^{C}{y}_{i}\cdot {\text{ln}}\left(\widehat{{y}_{i}}\right)$$

where $y$ is the true label for a sample, $\widehat{y}$ is the predicted probability distribution over the possible classes, and $C$ is the number of classes.

The categorical cross-entropy loss function is commonly used in multi-class classification problems like ours. The goal of the model is to predict the probability distribution over the possible classes, and the loss function measures the difference between the predicted distribution and the true distribution. The negative sign in the equation ensures that the loss is always a positive value. The smaller the value of the loss, the better the model is performing on the training data.

Explained variance is a metric that measures the proportion of the variation in the true values that is explained by the variance in the predicted values. It is a useful metric when evaluating the performance of a regression model, and provides insight into how well the model fits the data.

$$\mathrm{Explained\,Variance}=1-\frac{{\text{Var}}\left({\text{y}}-\widehat{{\text{y}}}\right)}{{\text{Var}}\left({\text{y}}\right)}$$

where $y$ is the true value and $\widehat{y}$ is the predicted value.

### Comparisons with prior studies

To assess the performance of various sleep stage classification models, we computed the overall precision, recall, and F1 score. Other metrics were listed in the source paper(s) and taken directly from there. In evaluating model performance across various sleep stages, we adopted a weighted approach to account for the uneven distribution of data among stages. Weighting ensures that stages with a higher frequency of actual or predicted occurrences exert a greater influence on the overall metrics, providing a more representative measure of the model's effectiveness. This is in contrast to a simple arithmetic mean, which could misrepresent performance by treating stages with disparate frequencies equally. Following this rationale, the overall precision and overall recall were computed as weighted averages, where the precision and recall of each stage were weighted by the recall and precision, respectively. This method offers a more holistic assessment of the model's performance across all sleep stages.

Overall precision was calculated as a weighted average of the precision for each sleep stage. The weight for each precision was given by the recall for that stage, thus:

$$\text{Overall Precision}=\frac{\text{Wake Precision}\times \text{Wake Recall}+\text{NREM Precision}\times \text{NREM Recall}+\text{REM Precision}\times \text{REM Recall}}{\text{Wake Recall}+\text{NREM Recall}+\text{REM Recall}}$$

Similarly, overall recall was computed as a weighted average of recall for each sleep stage. The weight for each recall was provided by the precision for that stage:

$$\text{Overall Recall}=\frac{\text{Wake Recall}\times \text{Wake Precision}+\text{NREM Recall}\times \text{NREM Precision}+\text{REM Recall}\times \text{REM Precision}}{\text{Wake Precision}+\text{NREM Precision}+\text{REM Precision}}$$

The F1 score, which provides a balance between precision and recall, was computed as the harmonic mean of the overall precision and recall:

$$\text{F1 Score}=\frac{2\times \text{Overall Precision}\times \text{Overall Recall}}{\text{Overall Precision}+\text{Overall Recall}}$$

These metrics were computed for each model, and the results were tabulated for comparison in Table 2.

### Computer hardware

We used a custom-built server for preprocessing and model training and testing:

- Intel i9-7980XE CPU @ 2.60 GHz (16-core)
- 128 GB System Memory (DDR4 3000 MHz)
- 3 × Nvidia Titan V (GV100) GPUs 12 GB VRAM each
- 2048 GB Samsung SSD 850

The server was running on Linux (Ubuntu 22.04 LTS), Nvidia Driver Version: 525.78.01, CUDA Version: 12.0, Python 3.10.10, Tensorflow 2.11.0.

### Supplementary Information

Supplementary Table 1.

## Data Availability

The preprocessed raw data, k-fold cross-validation models (and training metrics), and final model are available on Zenodo (10.5281/zenodo.7899654).

## Code availability

The code for preprocessing and model definition training and testing (including k-fold cross-validation) are available on Zenodo (10.5281/zenodo.7899654).
