# Supplementary material for: SlumberNet: deep learning classification of sleep stages using residual neural networks
Source: Sci Rep. 2024 Feb 27;14:4797. doi: 10.1038/s41598-024-54727-0 (PMC10899258; doi:10.1038/s41598-024-54727-0)
Supplement: Supplementary file 1 — Supplementary Table 1. [file 41598_2024_54727_MOESM1_ESM.pdf]

Supplementary Information

for

SlumberNet: Deep learning classification of sleep stages using residual neural networks

Pawan K. Jha[1,2,3#], Utham K. Valekunja[1,2,3#] & Akhilesh B. Reddy[1,2,3*]

[1] Department of Systems Pharmacology & Translational Therapeutics, Perelman School of Medicine, University of Pennsylvania, Philadelphia, PA 19104, USA.

[2] Institute for Translational Medicine and Therapeutics, Perelman School of Medicine, University of Pennsylvania, Philadelphia, PA 19104, USA.

[3] Chronobiology and Sleep Institute (CSI), Perelman School of Medicine, University of Pennsylvania, Philadelphia, PA 19104, USA.

[#] These authors contributed equally to the work

Supplementary Table 1

Detailed model summary of all layers in the SlumberNet model, their shapes, and the number of trainable parameters for each layer. A summary of the overall trainable and non-trainable parameter counts is given at the bottom of the table.

Layer (type)	Output Shape	Param #	Connected to
input_1 (InputLayer)	[(None, 256, 2, 1)]	0	[]
conv2d (Conv2D)	(None, 256, 2, 8)	136	['input_1[0][0]']
batch_normalization (BatchNorm alization)	(None, 256, 2, 8)	32	['conv2d[0][0]']
dropout (Dropout)	(None, 256, 2, 8)	0	['batch_normalization[0][0]']
activation (Activation)	(None, 256, 2, 8)	0	['dropout[0][0]']
conv2d_1 (Conv2D)	(None, 256, 2, 8)	648	['activation[0][0]']
batch_normalization_1 (BatchNo rmalization)	(None, 256, 2, 8)	32	['conv2d_1[0][0]']
dropout_1 (Dropout)	(None, 256, 2, 8)	0	['batch_normalization_1[0][0]']
activation_1 (Activation)	(None, 256, 2, 8)	0	['dropout_1[0][0]']
conv2d_3 (Conv2D)	(None, 256, 2, 8)	24	['input_1[0][0]']
conv2d_2 (Conv2D)	(None, 256, 2, 8)	392	['activation_1[0][0]']
batch_normalization_3 (BatchNo rmalization)	(None, 256, 2, 8)	32	['conv2d_3[0][0]']
batch_normalization_2 (BatchNo rmalization)	(None, 256, 2, 8)	32	['conv2d_2[0][0]']
add (Add)	(None, 256, 2, 8)	0	['batch_normalization_3[0][0]', 'batch_normalization_2[0][0]']
dropout_2 (Dropout)	(None, 256, 2, 8)	0	['add[0][0]']
activation_2 (Activation)	(None, 256, 2, 8)	0	['dropout_2[0][0]']
conv2d_4 (Conv2D)	(None, 256, 2, 16)	2064	['activation_2[0][0]']
batch_normalization_4 (BatchNo rmalization)	(None, 256, 2, 16)	64	['conv2d_4[0][0]']
dropout_3 (Dropout)	(None, 256, 2, 16)	0	['batch_normalization_4[0][0]']
activation_3 (Activation)	(None, 256, 2, 16)	0	['dropout_3[0][0]']
conv2d_5 (Conv2D)	(None, 256, 2, 16)	2576	['activation_3[0][0]']
batch_normalization_5 (BatchNo rmalization)	(None, 256, 2, 16)	64	['conv2d_5[0][0]']
dropout_4 (Dropout)	(None, 256, 2, 16)	0	['batch_normalization_5[0][0]']
activation_4 (Activation)	(None, 256, 2, 16)	0	['dropout_4[0][0]']
conv2d_7 (Conv2D)	(None, 256, 2, 16)	272	['activation_2[0][0]']
conv2d_6 (Conv2D)	(None, 256, 2, 16)	1552	['activation_4[0][0]']

```
batch_normalization_7 (BatchNo  (None, 256, 2, 16)  64          ['conv2d_7[0][0]']
rmalization)

batch_normalization_6 (BatchNo  (None, 256, 2, 16)  64          ['conv2d_6[0][0]']
rmalization)

add_1 (Add)                    (None, 256, 2, 16)   0           ['batch_normalization_7[0][0]',
                                                                 'batch_normalization_6[0][0]']

dropout_5 (Dropout)            (None, 256, 2, 16)   0           ['add_1[0][0]']

activation_5 (Activation)      (None, 256, 2, 16)   0           ['dropout_5[0][0]']

conv2d_8 (Conv2D)              (None, 256, 2, 32)   8224        ['activation_5[0][0]']

batch_normalization_8 (BatchNo  (None, 256, 2, 32)  128         ['conv2d_8[0][0]']
rmalization)

dropout_6 (Dropout)            (None, 256, 2, 32)   0           ['batch_normalization_8[0][0]']

activation_6 (Activation)      (None, 256, 2, 32)   0           ['dropout_6[0][0]']

conv2d_9 (Conv2D)              (None, 256, 2, 32)   10272       ['activation_6[0][0]']

batch_normalization_9 (BatchNo  (None, 256, 2, 32)  128         ['conv2d_9[0][0]']
rmalization)

dropout_7 (Dropout)            (None, 256, 2, 32)   0           ['batch_normalization_9[0][0]']

activation_7 (Activation)      (None, 256, 2, 32)   0           ['dropout_7[0][0]']

conv2d_11 (Conv2D)             (None, 256, 2, 32)   1056        ['activation_5[0][0]']

conv2d_10 (Conv2D)             (None, 256, 2, 32)   6176        ['activation_7[0][0]']

batch_normalization_11 (BatchN  (None, 256, 2, 32)  128         ['conv2d_11[0][0]']
ormalization)

batch_normalization_10 (BatchN  (None, 256, 2, 32)  128         ['conv2d_10[0][0]']
ormalization)

add_2 (Add)                    (None, 256, 2, 32)   0           ['batch_normalization_11[0][0]',
                                                                 'batch_normalization_10[0][0]']

dropout_8 (Dropout)            (None, 256, 2, 32)   0           ['add_2[0][0]']

activation_8 (Activation)      (None, 256, 2, 32)   0           ['dropout_8[0][0]']

conv2d_12 (Conv2D)             (None, 256, 2, 64)   32832       ['activation_8[0][0]']

batch_normalization_12 (BatchN  (None, 256, 2, 64)  256         ['conv2d_12[0][0]']
ormalization)

dropout_9 (Dropout)            (None, 256, 2, 64)   0           ['batch_normalization_12[0][0]']

activation_9 (Activation)      (None, 256, 2, 64)   0           ['dropout_9[0][0]']

conv2d_13 (Conv2D)             (None, 256, 2, 64)   41024       ['activation_9[0][0]']

batch_normalization_13 (BatchN  (None, 256, 2, 64)  256         ['conv2d_13[0][0]']
ormalization)

dropout_10 (Dropout)           (None, 256, 2, 64)   0           ['batch_normalization_13[0][0]']

activation_10 (Activation)     (None, 256, 2, 64)   0           ['dropout_10[0][0]']

conv2d_15 (Conv2D)             (None, 256, 2, 64)   4160        ['activation_8[0][0]']

conv2d_14 (Conv2D)             (None, 256, 2, 64)   24640       ['activation_10[0][0]']

batch_normalization_15 (BatchN  (None, 256, 2, 64)  256         ['conv2d_15[0][0]']
```

```
ormalization)

batch_normalization_14 (BatchN  (None, 256, 2, 64)  256         ['conv2d_14[0][0]']
ormalization)

add_3 (Add)                    (None, 256, 2, 64)   0           ['batch_normalization_15[0][0]',
                                                                 'batch_normalization_14[0][0]']

dropout_11 (Dropout)           (None, 256, 2, 64)   0           ['add_3[0][0]']

activation_11 (Activation)     (None, 256, 2, 64)   0           ['dropout_11[0][0]']

conv2d_16 (Conv2D)             (None, 256, 2, 128)  131200      ['activation_11[0][0]']

batch_normalization_16 (BatchN  (None, 256, 2, 128)  512        ['conv2d_16[0][0]']
ormalization)

dropout_12 (Dropout)           (None, 256, 2, 128)  0           ['batch_normalization_16[0][0]']

activation_12 (Activation)     (None, 256, 2, 128)  0           ['dropout_12[0][0]']

conv2d_17 (Conv2D)             (None, 256, 2, 128)  163968      ['activation_12[0][0]']

batch_normalization_17 (BatchN  (None, 256, 2, 128)  512        ['conv2d_17[0][0]']
ormalization)

dropout_13 (Dropout)           (None, 256, 2, 128)  0           ['batch_normalization_17[0][0]']

activation_13 (Activation)     (None, 256, 2, 128)  0           ['dropout_13[0][0]']

conv2d_19 (Conv2D)             (None, 256, 2, 128)  16512       ['activation_11[0][0]']

conv2d_18 (Conv2D)             (None, 256, 2, 128)  98432       ['activation_13[0][0]']

batch_normalization_19 (BatchN  (None, 256, 2, 128)  512        ['conv2d_19[0][0]']
ormalization)

batch_normalization_18 (BatchN  (None, 256, 2, 128)  512        ['conv2d_18[0][0]']
ormalization)

add_4 (Add)                    (None, 256, 2, 128)  0           ['batch_normalization_19[0][0]',
                                                                 'batch_normalization_18[0][0]']

dropout_14 (Dropout)           (None, 256, 2, 128)  0           ['add_4[0][0]']

activation_14 (Activation)     (None, 256, 2, 128)  0           ['dropout_14[0][0]']

conv2d_20 (Conv2D)             (None, 256, 2, 256)  524544      ['activation_14[0][0]']

batch_normalization_20 (BatchN  (None, 256, 2, 256)  1024       ['conv2d_20[0][0]']
ormalization)

dropout_15 (Dropout)           (None, 256, 2, 256)  0           ['batch_normalization_20[0][0]']

activation_15 (Activation)     (None, 256, 2, 256)  0           ['dropout_15[0][0]']

conv2d_21 (Conv2D)             (None, 256, 2, 256)  655616      ['activation_15[0][0]']

batch_normalization_21 (BatchN  (None, 256, 2, 256)  1024       ['conv2d_21[0][0]']
ormalization)

dropout_16 (Dropout)           (None, 256, 2, 256)  0           ['batch_normalization_21[0][0]']

activation_16 (Activation)     (None, 256, 2, 256)  0           ['dropout_16[0][0]']

conv2d_23 (Conv2D)             (None, 256, 2, 256)  65792       ['activation_14[0][0]']

conv2d_22 (Conv2D)             (None, 256, 2, 256)  393472      ['activation_16[0][0]']

batch_normalization_23 (BatchN  (None, 256, 2, 256)  1024       ['conv2d_23[0][0]']
ormalization)
```

```
 batch_normalization_22 (BatchN  (None, 256, 2, 256)  1024       ['conv2d_22[0][0]']
 ormalization)

 add_5 (Add)                    (None, 256, 2, 256)  0          ['batch_normalization_23[0][0]',
                                                                 'batch_normalization_22[0][0]']

 dropout_17 (Dropout)           (None, 256, 2, 256)  0          ['add_5[0][0]']

 activation_17 (Activation)     (None, 256, 2, 256)  0          ['dropout_17[0][0]']

 conv2d_24 (Conv2D)             (None, 256, 2, 256)  1048832    ['activation_17[0][0]']

 batch_normalization_24 (BatchN  (None, 256, 2, 256)  1024       ['conv2d_24[0][0]']
 ormalization)

 activation_18 (Activation)     (None, 256, 2, 256)  0          ['batch_normalization_24[0][0]']

 conv2d_25 (Conv2D)             (None, 256, 2, 256)  1048832    ['activation_18[0][0]']

 batch_normalization_25 (BatchN  (None, 256, 2, 256)  1024       ['conv2d_25[0][0]']
 ormalization)

 activation_19 (Activation)     (None, 256, 2, 256)  0          ['batch_normalization_25[0][0]']

 conv2d_26 (Conv2D)             (None, 256, 2, 256)  1048832    ['activation_19[0][0]']

 batch_normalization_27 (BatchN  (None, 256, 2, 256)  1024       ['activation_17[0][0]']
 ormalization)

 batch_normalization_26 (BatchN  (None, 256, 2, 256)  1024       ['conv2d_26[0][0]']
 ormalization)

 add_6 (Add)                    (None, 256, 2, 256)  0          ['batch_normalization_27[0][0]',
                                                                 'batch_normalization_26[0][0]']

 dropout_18 (Dropout)           (None, 256, 2, 256)  0          ['add_6[0][0]']

 activation_20 (Activation)     (None, 256, 2, 256)  0          ['dropout_18[0][0]']

 global_average_pooling2d (Glob  (None, 256)          0          ['activation_20[0][0]']
 alAveragePooling2D)

 dense (Dense)                  (None, 3)            771        ['global_average_pooling2d[0][0]'
                                                                ]

==================================================================================================
Total params: 5,345,011
Trainable params: 5,338,931
Non-trainable params: 6,080
__________________________________________________________________________________________________
```
